# Taxonomic Status and Phylogenetic Relationship of the Charadriidae Family Based on Complete Mitogenomes

**DOI:** 10.2174/0113892029273517231017051819

**Published:** 2023-12-12

**Authors:** Weiya Qian, Yizheng Liu, Keer Miao, Qing Chang, Chaochao Hu

**Affiliations:** 1 Jiangsu Key Laboratory for Biodiversity and Biotechnology, School of Life Sciences, Nanjing Normal University, Nanjing, Jiangsu, China;; 2 Analytical and Testing Center, Nanjing Normal University, Nanjing, Jiangsu, China

**Keywords:** *Charadrius leschenaultii*, *Charadrius mongolus*, comparative mitogenomics, phylogenetics, Charadriidae, relationship

## Abstract

**Background:**

The Charadriiformes provide a good source for researching evolution owing to their diverse distribution, behavior, morphology, and ecology. However, in the Charadrii, family-level relationships remain understudied, and the monophyly of Charadriidae is also a subject of controversy.

**Methods:**

In the present study, we generated complete mitogenomes for two species, *Charadrius leschenaultii* and *Charadrius mongolus*, which were found to be 16,905 bp and 16,844 bp in length, respectively. Among the 13 protein codon genes, we observed variation in the rate of non-synonymous substitution rates, with the slowest rate found in COI and the fastest rate observed in ATP8. The Ka/Ks ratio for all Charadriidae species was significantly lower than one, which inferred that the protein-coding genes underwent purifying selection.

**Results:**

Phylogenetic analysis based on the genes of Cyt *b*, 12S and ND2 revealed that the genus *Pluvialis* is the sister group of three families (Haematopodidae, Ibidorhynchidae, Recurvirostridae). However, the phylogenetic analysis based on complete mitogenomes indicated that the genus *Pluvialis* is within the Charadriidae family.

**Conclusion:**

This study highlights the importance of carefully selecting the number of genes used to obtain accurate estimates of the species tree. It also suggests that relying on partial mtDNA genes with fast-evolving rates may lead to misleading results when resolving the *Pluvialis* sister group. Future research should focus on sequencing more mitogenomes at different taxonomic levels to gain a better understanding of the features and phylogenetic relationships within the Charadriiformes order.

## 1 INTRODUCTION

The birds Charadriiformes are a diverse order that can be taxonomically divided into three main clades, Lari (buttonquails, allies, and their auks, along with gulls) is a sister group to Scolopaci (allies, jacanas, and sandpipers) whilst Charadrii (plovers, oystercatchers and allies) is placed at the base [1-4]. The variation in morphological traits, behavioral ecology, and ecological relationships makes Charadriiformes a popular subject to study [5-7]. The advancements in molecular sequencing technologies, such as the next-generation sequencers, and the development of new analytical methods have improved the study of the relationships among many species of Charadriiformes [8-11]. However, despite the increased sampling of loci and taxa, the position of the ibisbill (Ibidorhyncha), and the relationships among their five subfamilies of the gulls (Laridae) still have not been fully resolved [5, 12].

Within the Charadrii clade, the relationships at the family level based on morphological and molecular data are not well studied [1, 13]. The monophyly of lapwings and plovers (Charadriidae) is also controversial [14]. In molecular phylogenies, the plovers are surprisingly recovered as a paraphyletic group, with the Black-bellied Plover (*Pluvialis squatarola* Linnaeus, 1758) being a sister species to a clade containing the morphologically disparate oystercatchers, stilts, and avocets, or placed as a sister to all other Charadrii [5, 12, 13, 15, 16]. Ancestral area analyses using parsimony and Bayesian phylogenetic methods suggest that *Charadrius* plovers originated in the Northern Hemisphere, with major radiations in this group linked to changes in the range of ancestral plover species that resulted in colonization of the Southern Hemisphere [17]. The Charadriidae family records 12 genera and 70 species worldwide [18], with 18 species recorded in China alone [19]. The variation in morphological traits, behavioral, ecological, and macroevolutionary processes makes Charadriidae a popular subject of study [16, 20-22].

Animal mitogenome is an approximately 16 kb circular molecule [23]. A typical metazoan mitogenome consists of 22 transfer RNA genes (tRNAs), two ribosomal RNA genes (rRNAs), 13 protein-coding genes (PCGs), and one non-coding region (CR) [24-26]. Due to its characteristics such as maternal inheritance, a low rate of genetic recombination, and small genome size, the mitogenome is an important research tool for population genetics, molecular systematics, phylogeography, and phylogenetics [11, 27-30]. Additionally, Cyt *b*, 12S rRNA, and ND2 are widely used in phylogenetic studies. The gene of 12S rRNA is highly conserved and has been used as a molecular marker for molecular phylogeny [31]. Previous studies recovered a strongly supported clade comprising Laridae, Sternidae and *Rynchops* using the combination genes of COI, ND2 and Cyt *b* [32]. The relationships among species in Rallidae were examined using the genes of COI and Cyt *b* [33]. Gibson and Baker (2012) used five genes (RAG1, Cyt *b*, 12S, ND2, and COI) to recover the phylogenetic relationships in the Scolopaci [34].

To date, ten complete mitogenomes of Charadriidae (five species of *Charadrius*, three of *Vanellus,* and two of *Pluvialis*) are available in GenBank (http://www.ncbi.nlm.nih.gov) (Table **1**) [35-42]. The limited number of Charadriidae complete mitogenomes restricts the exploration of phylogenetic relationships and evolutionary patterns in this family. In this study, we obtained the complete mitogenome of *Charadrius leschenaultii* Lesson, 1826 and *Charadrius mongolus* Pallas, 1776 in Charadriidae. We combined these newly sequenced mitogenomes with previously sequenced ten complete mitogenomes from the family of Charadriidae available in GenBank to perform a detailed comparative mitogenomic analysis of Charadriidae species. Additionally, we reconstructed relationships at the family level within Charadrii by combining 37 mitogenomes of Cyt *b*, 12S and ND2 in Charadrii. Through this study, we aim to provide new insights into the phylogeny and evolution of Charadrii.

## 2 MATERIALS AND METHODS

### 2.1 Sampling and DNA Extraction

The specimen of *C. leschenaultii* and *C. mongolus* was obtained from a derelict mist net in Xiaoyangkou, Nantong City, Jiangsu Province, China (32°33′18.74″N, 120°3′0. 39″E), being both already dead. The bird species were identified by morphological method, referring to ‘A Checklist on the Classification and Distribution of the Birds of China (Third Edition)’ [19]. They were collected in July 2019, and two muscle samples were preserved in 95% ethanol and stored at −20°C in our laboratory. The specimens were deposited in Nanjing Normal University under voucher numbers of NJNU-Cles08 and NJNU-CMon05. Total genomic DNA was extracted using standard phenol-chloroform procedures [42]. The concentration of DNA was determined by 260/280 using a Nanodrop 1000 Spectrophotometer (Thermo Scientific, Waltham, MA, USA). The quality of DNA was assessed through electrophoresis in a 1% agarose gel.

### 2.2 Library Preparation and Sequencing

Utilizing an ultrasonic process, whole genomic DNA was cut to 400 - 600 bp and delivered to Novogene (Beijing, China) for sequencing. Following the manufacturer’s instructions, the sequencing libraries were prepared using the Illumina TruSeq DNA Sample Preparation Kit (Illumina, San Diego, CA, USA) and, then, sequenced as PE 2 × 150 bp onto the Illumina Novaseq 6000 platform (Illumina, San Diego, CA) at Novogene (Beijing, China). Fastp v. 0.21 was used to filter raw data into clean reads prior to *de novo* assemblies [43]. Geneious 10.1.2 was used to assemble clean reads [44]. The *Charadrius alexandrinus* Linnaeus, 1758 (GenBank no. MF565382) was used as a reference map when assembling because the *C. alexandrinus* was a closely related species to the *C. leschenaultii* and *C. mongolus*. The obtained complete mitogenomes for sand plovers from Charadriidae built herein were deposited in GenBank (Accession No. ON950055 and ON986363).

### 2.3 Mitogenome Annotation and Sequence Analysis

By selecting the vertebrate mitochondrial genetic code, the sites of the PCGs were recognized using ORF Finder *via* NCBI and MITOS [45], tRNAscan-SE 2.0 [46], and ARWEN [47] were used to recognize and annotate the tRNA and rRNA genes. The mitochondrial genetic maps of two species were produced by the OGDRAW program (https://chlorobox.mpimp-golm.mpg.de/OGDraw.html) [48]. Strand asymmetry of the mitogenome was calculated according to the following formulas: AT skew = (A – T)/ (A + T) and GC skew = (G − C)/ (G + C) [49, 50]. Analysis of nucleotide composition in different regions and codon usage were performed using MEGA X software [51]. Non-synonymous (Ka, π modified) and synonymous (Ks, π modified) substitution rates in 13 PCGs were calculated in DnaSP 5.1 [52].

### 2.4 Phylogenetic Analysis

Phylogenetic relationships were assessed by Bayesian inference (BI) and maximum likelihood (ML) with two concatenated gene sets: (a) Cyt *b*, 12S and ND2 (2,495 bp) of 37 species; (b) 13 PCGs + 2 rRNAs (14,136 bp) of 20 complete mitogenomes from Charadrii were produced for phylogenetic reconstruction. Two species (*Columba livia* Gmelin, 1758. GenBank no. GU908131 and *Gallus gallus* Linnaeus, 1758. GU261719) were used as outgroups [53, 54]. In PartitionFinder 2, the greedy algorithm and Akaike information criteria (AICc) were used to calculate the best partitioning scheme and optimality criterion to indicate the most appropriate nucleotide evolution model for each partition [55]. In BI analysis, we made two simultaneous runs of 1.0 × 10^6^ generations, sampling every 1000 generations of trees, with three heated and one cold chain to encourage swapping among the Markov-chain Monte Carlo (MCMC) chains. In addition, the average standard deviation of split frequencies for the two runs was examined (< 0.01). After discarding the first 25% of trees as a “burn-in” step, the 50% majority rule consensus of the post-burn-in trees sampled at stationarity was used to derive the Bayesian posterior probability in two gene sets. The BI method was performed using MrBayes v3.2.2 [56]. The maximum likelihood (ML) analysis was conducted by RAxML 8.1.17 [57] using the GTR + G model, a quick bootstrapping set with 10 runs and 1,000 replications was used to evaluate branch support. The tree information was visualized and edited by FigTree v. 1.4.4 [58].

## 3 RESULTS AND DISCUSSION

### 3.1 Genome Organization

**Fig. (1):**
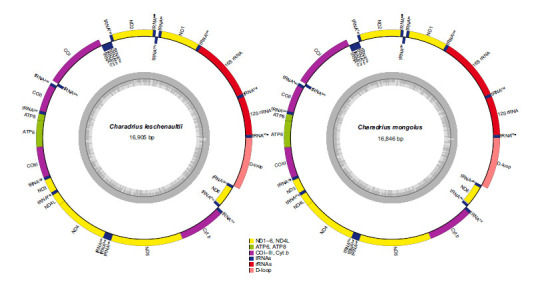
Mitogenome maps of *Charadrius leschenaultii* Lesson, 1826 and *Charadrius. mongolus* Pallas, 1776. The inner dark gray indicates GC content. Yellow represents NADH dehydrogenase. Green represents ATP synthase. Purple indicates COI, COII, COIII, and Cyt *b*. Dark blue indicates tRNAs. Red represents rRNAs. Pink represents the D-loop.

The complete mitogenome of *C. leschenaultii* and *C. mongolus* was 16,905 bp and 16,844 bp in length, respectively. They each were composed of 37 genes and a non-coding region (Fig. **1**). One of the 13 PCGs (ND6) and eight tRNA genes (tRNA^Cys^, tRNA^Tyr^, tRNA^Ala^, tRNA^Asn^, tRNA^Pro^, tRNA^Ser^, tRNA^Glu^ and tRNA^Gln^) were located on the minus strand, and the other 12PCGs, 14tRNAs, 2rRNAs were located on the plus strand. Gene order is conserved across all species and the same as that of other Charadriidae, showing no structural rearrangement [59]. The 12 complete Charadriidae species mitogenomes range in size from 15,933 bp (*Charadrius dubius*) to 17,135 bp (*Vanellus cinereus*), with an average size of 16,827 bp (SD = 286.34, n = 12) (Table **1**). In PCGs, tRNAs, and rRNAs, the length variation was minimal; however, most of the size variation was mainly caused by mutations in the CR. The intergenic spacers span 1-23 bp in length in the *C. leschenaultii* and *C. mongolus*, with the longest one being sited between the tRNA^Tyr^ and tRNA^Pro^ genes.

### 3.2 Mitochondrial Genomic Composition

The overall mean base composition of Charadriidae mitogenomes was listed as follows: A, 31.41%; C, 31.06%; T, 23.72% and G, 13.74%. The proportion of A + T was slightly higher than that of G + C in twelve Charadriidae mitogenomes. AT rich ranged from 54.30% (*Pluvialis squatarola*) to 55.58% (*Charadrius vociferous*) (Table **1**), which is consistent with previous avian mitogenomes [9, 60]. Additionally, the content of higher A + T is found not only in the CR but also in PCGs and rRNAs. The mean AT skew value was 0.14 ± 0.01 (mean ± SD), ranging from 0.13 (*Vanellus vanellus* and *Charadrius mongolus* GenBank no. ON 986363) to 0.15 (*Vanellus cinereus* GenBank no. KM873665, *Pluvialis fulva* and *Charadrius mongolus* GenBank no. MW298528). The GC skew value ranged from −0.40 (*Charadrius vociferous*) to −0.37 (*Charadrius mongolus* GenBank no. MW298528). All mitogenomes exhibited A-skew and obvious C-skew, which was consistent with findings in other birds [61-63]. However, a significant negative AT skew was in ND3 in *Charadrius vociferous* on the P-strand.

### 3.3 The Evolutionary Rate of Protein-coding Genes

Taking out termination codons and indels, the lengths of protein-coding genes in each species totaled 11,322 bp. Comparison of each PCG among Charadriidae species provides a better understanding of gene evolution. The length of 13 PCGs varied from 165 bp (ATP8) to 1,812 bp (ND5). COIII gene was found to have the slowest percentage of variable positions (26.95%), while the ATP8 gene was found to have the highest percentage of variable positions (46.67%) and parsimony informative sites (32.73%), indicating that this gene included more variable sites than the other PCGs (Table **2**).

The average uncorrected pairwise distances (Aupd) revealed the heterogeneity of evolutionary rate for each gene, the COI gene (0.01) and COII gene (0.02) were slow, whereas those of ND2 (0.09) and ATP8 (0.18) were considerably fast. So, we can infer that ATP8 gene was the least conserved PCG, while COI gene was the most conserved. To further investigate the role of selection and better understand the evolution at the DNA level in Charadriidae species, Ka, Ks and Ka/Ks were calculated for each PCG, respectively. The slowest average value of Ka was observed in the gene COI (0.01), while the ATP8 gene was the highest average value of Ka (0.1) and Ks (0.62). Average Ka/Ks was observed to be lower than one in all protein-coding genes across all species, indicating purifying selection, and to be highest for the ATP8 gene (Ka/Ks = 0.16).

### 3.4 The Codon Usage

Start and stop codons are different in the PCGs from the family Charadriidae, and generally ATG and TAA were the most frequently used. Five types of conventional vertebrate mitochondrial start codon (ATG, GTG, ATA, ATC and ATT) were found in the 13 PCGs. ATG was the most frequently used start codon, accounting for 76.28% of the total start codons, followed by GTG (15.38%). Except for COI, ND3 and ND5, which initiate with the alternative codons GTG, ATC and GTG respectively, ten of the 13 PCGs start with normal ATG start codon. Nine genes (ND6, Cyt *b*, ND4, ND4L, COIII, ATP6, ATP8, COII and ND2) only stared at ATG.

PCGs end in complete TAA, TAG, AGG, AGA or AAA codons or with incomplete TA– or T–, which are thought to be completed by posttranscriptional modifications like polyadenylation [64-67]. Only seven genes (Cyt *b*, ND4L, ND3, ATP6, ATP8, COII and ND6) used the most frequent codon, TAA, as a stop codon. In the case of other bird orders, the stop codon TAA appears only in Cyt *b*, ND4L and COII [68]. AGG appeared in ND1and COI. The genes of ND2, ND5 and ND6 stopped with TAG. AGA and AAA were discovered only in ND5 and ND3, respectively. The incomplete stop codon T found in ND2, COIII and ND4 was similar with other avian mitogenomes [69, 70].

**Fig. (2):**
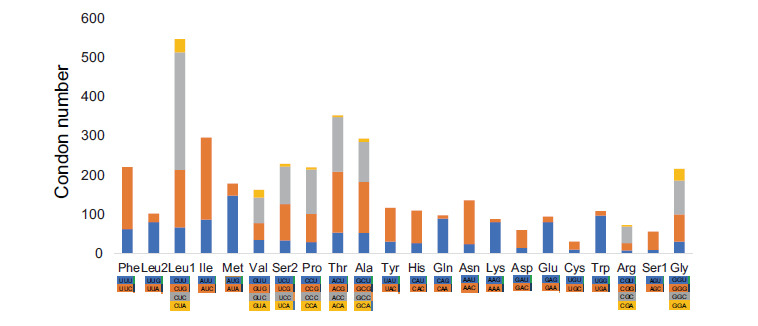
The value of relative synonymous codon usage of the species mitogenomes in the family charadriidae, excluding stop codons.

**Fig. (3):**
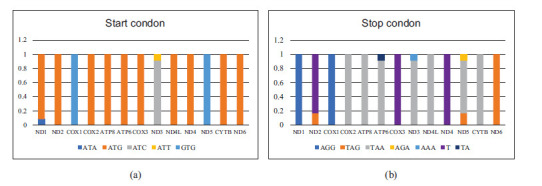
Usage of start (**a**) and stop (**b**) codons in the 13 protein-coding genes of family charadriidae.

The pattern of codon usage was influenced by the nucleotide composition bias (Figs. **2** and **3**). Among the 62 amino acid encoding codons, Leu^CUA^, Ile^AUC^, and Phe^UUC^ were the most frequently used and the least was Arg^CGG^ (0.09%).

### 3.5 Phylogenetic Analysis

**Fig. (4):**
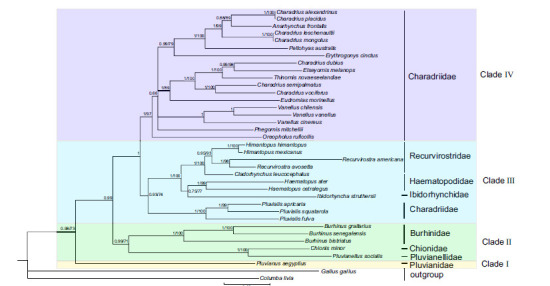
The phylogenetic tree was derived from a gene set (concatenated by Cyt *b*, 12S, and ND2) using BI analysis and ML analysis. Numbers left to nodes represent posterior probabilities for BI /bootstrap supports for ML. The colors designate the clades. Yellow represents Clade I. Green represents Clade II. Blue represents Clade III. Purple represents Clade IV.

Phylogenetic analysis (BI and ML) based on of Cyt *b*, 12S and ND2 (Dataset a with 2,495 bp) for 37 species converged to the same topology. The posterior probability values for all the nodes were very high. The topologies constructed recovered four major clades among Charadrii (Clade I, II, III and IV; Fig. **4**). *Pluvianus aegyptius* forms a single root clade. In clade III, the genus *Pluvialis* forms the sister group of three families Haematopodidae, Ibidorhynchidae, Recurvirostridae. The clade IV grouped the remaining species. Within clade IV, *C. leschenaultii* is a sister species of *C. mongolus* with a high nodal support value. Besides, the positioning of genera *Elseyornis*, *Thinornis*,*Anarhynchus*, *Phegornis*, and *Vanellus* is within the genus *Charadrius* clade, which is in agreement with the previous study based on eight mitochondrial and two nuclear markers, the monophyly of this clade is controversial [20].

It is worth noting that there was a close genetic relationship between *C. alexandrines* and *C. placidus*. The main conflict involved the relationships among the genus *Pluvialis* (Fig. **4**). It is supported that the genus *Pluvialis* is close to the Haematopodidae, Ibidorhynchidae, and Recurvirostridae, contradicting the traditional morphological hypothesis of Charadriidae monophyly [65] as well as the nuclear sequence analysis [15, 71]. However, the phylogenies recovered the genus *Pluvialis* within the Charadriidae based on complete mitogenomes [5, 16, 72], whereas the taxonomically comprehensive analysis supported plover paraphyly by three nuclear loci in addition to mtDNA data [73].

**Fig. (5):**
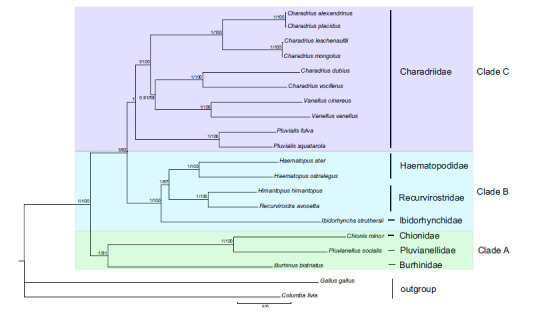
The phylogeny tree derived from 13 PCGs and two rRNAs combined sequences among Charadrii. Numbers left to nodes represent posterior probabilities for BI/bootstrap supports for ML. Green represents Clade A. Blue represents Clade B. Purple represents Clade C.

Combined with 13 mitochondrial PCGs, 12S and 16S genes, the Dataset b was 14,136 bp long after alignment. The BI and ML recovered three main clades (Clade A, B, and C; (Fig. **5**). Clade A included three families, with Burhinidae forming a sister group of two families (Chionidae and Pluvianellidae). The family Ibidorhynchidae was also the sister group with two families Haematopodidae and Recurvirostridae in clade B. Clade C grouped the remaining species which were all in Charadriidae, and the genus *Pluvialis* was identified as the most basal position within the family Charadriidae in agreement with previous research [5, 9, 14]. The newly described mitogenome will be valuable for studies on shorebird's molecular systematics and phylogenetics in the future, as well as for disentangling the taxonomy within plovers.

## CONCLUSION

In this study, we sequenced and annotated the mitogenome of two species of Charadriidae (*C. leschenaultii* and *C. mongolus*). The mitogenomes exhibited typical genome organization and gene order. The base composition in 12 species from Charadriidae consistently biased towards AT-rich. The average uncorrected pairwise distances revealed heterogeneity in the evolutionary rate for each gene, indicating that the least conserved PCG was ATP8, while the most conserved was COI. The Ka/Ks radio suggested that the PCGs were undergoing purifying selection. Based on the genes of Cyt *b*, 12S and ND2, the phylogenetic analysis found that the genus *Pluvialis* forms the sister group of three families ((Haematopodidae, Ibidorhynchidae), Recurvirostridae). However, the phylogenetic tree based on complete mitogenomes revealed that *Pluvialis* was within the Charadriidae. The trees also demonstrated that *C. leschenaultii* was a sister species to *C. mongolus*. The molecular data obtained in this study provide useful insights into the phylogeny and evolution of the Charadriidae. In the future, more mitogenomes at different taxonomic levels will be sequenced, providing useful information for a better understanding of the features and phylogenetic relationship of Charadriiformes.

## Figures and Tables

**Table 1:** Nucleotide composition and strand asymmetry of mitogenomes in 12 species. Mitogenomes that were newly sequenced are characterized by an “*”.

**Species**	**Length(bp)**	**GenBank**	**A+T%**	**AT-skew**	**GC-skew**
Charadrius leschenaultii*	16,905	ON950055	55.56	0.15	–0.37
Charadrius mongolus*	16,844	ON986363	55.39	0.13	–0.39
*Pluvialis squatarola*	16,860	MT561267	54.30	0.14	–0.38
*Vanellus cinereus*	17,074	KM404175	55.15	0.15	–0.39
*Vanellus cinereus*	17,135	KM873665	55.14	0.14	–0.38
*Vanellus vanellus*	16,795	KM577158	55.47	0.13	–0.38
*Pluvialis fulva*	16,854	KX639757	54.87	0.15	–0.39
*Charadrius placidus*	16,895	KY419888	55.16	0.14	–0.39
*Charadrius alexandrinus*	16,905	MF565382	55.24	0.14	–0.39
*Charadrius vociferous*	16,808	MN356113	55.58	0.14	–0.40
*Charadrius dubius*	15,933	OM063155	54.99	0.14	–0.39
*Charadrius mongolus*	16,919	MW298528	55.47	0.14	–0.39

**Table 2:** The composition and variation analysis of 13 protein-coding genes in charadriidae.

**Genes**	**Length (bp)**	**AT%**	**AT- skew**	**GC- skew**	**%Vs**	**%Pis**	**%S**	**ts/tv**	**Ks**	**Ka**	**Ka/Ks**	**Aupd**
ND1	975	55.37	0.04	−0.43	32.82	25.95	6.87	4.43	0.64	0.02	0.03	0.03
ND2	1038	57.38	0.15	−0.56	36.71	25.24	11.46	3.99	0.54	0.05	0.09	0.09
COI	1548	52.90	0.05	−0.32	27.2	19.96	6.65	4.65	0.52	0.01	0.02	0.01
COII	681	54.48	0.11	−0.36	29.07	21.44	7.64	4.76	0.54	0.02	0.04	0.02
ATP8	165	57.12	0.19	−0.73	46.67	32.73	13.94	3.9	0.62	0.1	0.16	0.18
ATP6	681	53.82	0.09	−0.57	32.16	24.96	7.2	3.46	0.53	0.03	0.06	0.05
COIII	783	53.64	0.07	−0.35	26.95	20.82	6.13	4.49	0.53	0.01	0.02	0.02
ND3	348	56.27	0.05	−0.48	31.32	22.41	8.91	3.6	0.51	0.04	0.08	0.06
ND4L	294	53.12	0.07	−0.42	32.99	24.49	8.5	4.58	0.58	0.03	0.05	0.05
ND4	1374	55.25	0.16	−0.56	33.99	23.65	10.33	3.65	0.47	0.04	0.09	0.07
ND5	1812	55.31	0.18	−0.50	38.02	23.84	14.18	2.67	0.52	0.05	0.1	0.09
Cyt *b*	1110	53.45	0.05	−0.48	34.5	21.62	12.88	2.92	0.56	0.03	0.05	0.05
ND6	513	53.30	0.60	−0.57	38.79	27.29	11.5	4.19	0.52	0.06	0.12	0.09

Note: Vs: variable sites, Pis: parsimony informative sites, S: singleton, ts/tv: the estimated transition/transversion bias, Aupd: The average uncorrected pairwise distances.

## Data Availability

The datasets generated and/or analyzed during the current study are available in the GenBank repository, accession numbers ON950055 and ON986363.

## LIST OF ABBREVIATIONS

AICc: Akaike Information Criteria
BI: Bayesian Inference
MCMC: Markov-chain Monte Carlo
ML: Maximum Likelihood
PCGs: Protein-coding Genes
rRNAs: ribosomal RNA Genes
tRNAs: transfer RNA Genes
